# Preslaughter diet management in sheep and goats: effects on physiological responses and microbial loads on skin and carcass

**DOI:** 10.1186/2049-1891-5-42

**Published:** 2014-08-28

**Authors:** Govind Kannan, Venkat R Gutta, Jung Hoon Lee, Brou Kouakou, Will R Getz, George W McCommon

**Affiliations:** 1 Agricultural Research Station, Fort Valley State University, 1005 State University Drive, Fort Valley, GA 31030, USA

**Keywords:** *E. coli* contamination diet, Goats, Physiology, Sheep

## Abstract

Sixteen crossbred buck goats (Kiko x Spanish; BW = 32.8 kg) and wether sheep (Dorset x Suffolk; BW = 39.9 kg) were used to determine the effect of preslaughter diet and feed deprivation time (FDT) on physiological responses and microbial loads on skin and carcasses. Experimental animals were fed either a concentrate (CD) or a hay diet (HD) for 4 d and then deprived of feed for either 12-h or 24-h before slaughter. Blood samples were collected for plasma cortisol and blood metabolite analyses. *Longisimus* muscle (LM) pH was measured. Skin and carcass swabs were obtained to assess microbial loads. Plasma creatine kinase activity (863.9 and 571.7 ± 95.21 IU) and non-esterified fatty acid concentrations (1,056.1 and 589.8 ± 105.01 mEq/L) were different (*P* < 0.05) between sheep and goats. Species and diet treatments had significant effects on the ultimate pH of LM. Pre-holding total coliform (TCC) and aerobic plate counts (APC) of skin were significantly different between species. Goats had lower (*P* < 0.05) TCC (2.1 vs. 3.0 log_10_ CFU/cm^2^) and APC (8.2 vs. 8.5 log_10_ CFU/cm^2^) counts in the skin compared to sheep. Preslaughter skin *E. coli* counts and TCC were different (*P* < 0.05) between species. Goats had lower (*P* < 0.05) counts of *E. coli* (2.2 vs. 2.9 log_10_ CFU/cm^2^) and TCC (2.3 vs. 3.0 log_10_ CFU/cm^2^) in the skin compared with those in sheep. Diet, species, and FDT had no effect (*P* > 0.05) on *E. coli* and TCC in carcass swab samples. The APC of carcass swab samples were only affected (*P* < 0.05) by the FDT. The results indicated that preslaughter dietary management had no significant changes on hormone and blood metabolite concentrations and sheep might be more prone for fecal contamination than goats in the holding pens at abattoir.

## Background

The hide and viscera of animals entering the abattoir are potential sources of contamination of carcasses with pathogenic bacteria [1]. The hide of the live animal becomes contaminated with pathogenic and non-pathogenic microorganisms from a wide range of sources such as feces, soil, water, and vegetation [2]. Animals can spread the contaminants to other animals during preslaughter transport and holding, directly via physical contact with one another or with the contaminated floor [3]. Fecal shedding of bacteria can be controlled by manipulating the preslaughter diet [4] and feed deprivation time [5] in ruminants.

Preslaughter dietary manipulation may not only affect the micro flora in gastrointestinal tracts in ruminants, but may also influence the variables related to meat quality and animal welfare [6,7]. Feeding grain diets can change the rumen and intestinal microbial populations [8]. Overfeeding cattle with grain has been shown to cause a 2 log scale increase in total coliform counts [9].

Stress and dehydration resulting from preslaughter management methods can adversely affect production variables such as live and carcass weights as well as meat quality [10]. A switch to hay feeding from a concentrate diet is likely to influence carcass weights, although Stanton and Schultz [11] indicated that such a diet change did not have a dramatic impact on carcass characteristics and final body weights in cattle. However, Kannan et al. [12] reported that 18 h of feed deprivation resulted in a 10% live weight shrinkage in goats. Earlier studies also showed that fasting sheep for 24 h resulted in about 7% live weight loss due to reduction in gut contents [13,14]. Feed deprivation is one of the preslaughter stress factors that may be responsible for depletion of muscle glycogen prior to slaughter [15]. Preslaughter depletion of muscle glycogen may result in an abnormally high pH of meat, which may have adverse effects on meat quality such as dark cutters [16] and poor shelf life due to microbial spoilage [17].

Blood hormone and metabolites in ruminants are also influenced by feed deprivation. Plasma cortisol concentration, a good indicator of welfare status during the preslaughter period in food animals [18], increases in sheep [19] and goats [12] due to feed deprivation. Feed deprivation also alters plasma glucose [20,21], urea nitrogen [10,12], and non-esterified fatty acid [22,23]. Kannan et al. [24] reported an increase in creatine kinase activity in the circulation during preslaughter feed deprivation in goats.

The objectives of this study were, therefore, to estimate the efficacy of preslaughter diet (concentrate vs roughage) and feed deprivation time (12 vs. 24 h) on *E. coli* and other enteric bacterial population on skin and carcass, as well as to determine the effects on blood hormone and metabolites in sheep and goats.

## Methods

### Animal feeding and feed deprivation treatments

Experimental procedures involving animals were conducted with approval of the Fort Valley State University (FVSU) Institutional Animal Care and Use Committee. Animals were obtained from the Georgia Small Ruminant Research and Extension Center at (FVSU). Sixteen crossbred wether sheep (Dorset x Suffolk; BW = 39.9 ± 0.88 kg) and buck goats (Kiko x Spanish; BW = 32.8 ± 0.91 kg) grazed on winter pea and rye grass dominant forages were assigned in a completely randomized design to a feeding trial consisting of two dietary treatments: primarily corn based concentrate (Table 1) and Bermuda grass hay diets. Each treatment was replicated in two pens with either four sheep or goats per pen. Each pen of four experimental animals was fed twice a day either a concentrate (CD) or hay diet (HD) with ad libitum access to water for 4 days. At the end of the 4-d feeding trial, half of animals from each pen (n = 16) were randomly selected and transported to the university slaughter and processing facility. Each animal was weighed and then assigned to a pen in the holding area according to the original pen numbers in order to maintain the same social group. This group of animals was deprived of feed for a 24-h period with continuous access to water. Other half of animals (n = 8/pen) were assigned to deprive of feed for a 12 h period according to previously descried in the 24 h feed deprivation. Both feed deprivation time (FDT) groups were processed on the same day such that harvest occurred within the same time frame for both group.

**Table 1:** **Ingredient composition of concentrate diet**^**1,2 **^**fed to sheep and goats**

**Ingredient**	**Composition,%**
Cottonseed hull	14.0
Ground corn	67.8
Soybean meal	13.6
Poultry fat	2.73
Trace minerals^3^	0.5
Vitamin premixed	0.5
Dicalcium phosphate	0.9

^1^Predicted digestible Energy (DE) = 4.0 Mcal/kg.

^2^Crude protein = 12.9%.

^3^Composition: NaCl, 45 to 50%; Ca 9.0 to 10.8%; P, >4.5%; Mg, >1.5%; K, >0.9%; S, >0.3%; Zn, >1.55%; and I, >180 ppm; Fe, >2,000 ppm; Mn, >4,000 ppm; Se, >60 ppm; vitamin A, >2,200,000 IU; vitamin D_3_, >165,000 IU; and vitamin E, >6,600 IU/kg.

#### *Animal behavior*

Behavior of each animal was monitored for a 90-min period before slaughter. The weather conditions were identical on the experimental days. Minimum temperatures ranged from 5 to 7°C and maximum temperatures ranged from 18 to 20°C. Standing, moving, agonistic (ramming, jumping, horning, and head butting) lying and drinking behaviors were recorded. Behavioral observations were made every minute using the scan sampling method in each pen (from pen 1 to 8) [25]. At each monitoring period, the number of animals performing each behavior was recorded. Animals were slaughtered in a predetermined order and rotated among pens to avoid confounding of effects.

### Blood sampling and analysis

Blood samples were collected from each animal at the beginning of the feeding trial (pretrial) and prior to slaughter. Blood samples were collected by trained personnel via jugular venipuncture into 10 mL Vacutainer tubes containing 81 μL of 15% EDTA solution and immediately placed on ice. All efforts were made not to agitate the animals during sampling. Plasma was separated by centrifugation at 1,000 × g for 30 min in a Sorvall Superspeed model 5RC2-B automatic refrigerated centrifuge (Ivan Sorvall Inc., Newton, CT) and stored in a 10-mL vial at -20°C for determination of plasma cortisol, glucose, creatine kinase (CK), urea nitrogen (PUN), and non-esterified fatty acid (NEFA) concentrations.

Plasma cortisol concentrations were determined using a Coat-A-Count radioimmunoassay (RIA) kit (Diagnostic Product Corp., Los Angeles, CA) as described by Kannan et al. [12]. Blood glucose and PUN concentrations and CK activity were analyzed using an IDEXX VetTest® instrument (IDEXX Laboratories Inc., Westbrook, ME). The plasma sample was delivered into a pipette tip and dispensed onto each metabolite testing slide. As the sample was absorbed and filtered through the layers of the slides, color changes occurred due to biochemical reactions. The color and intensity were measured by an optical system. Plasma NEFA concentrations were analyzed using a commercially available kit (Wako Chemicals, Richmond, VA) as described by Kannan et al. [24]. The assay was performed using acetyl CoA synthetase/acetyl-CoA oxidase method (NEFA C Code No. 994–75409 E). The absorbance values were determined using a Shimadzu® (Model UV-2401 PC) UV–VIS spectrophotometer (Shimadzu Scientific Instruments, Inc., Columbia, MD).

### Carcass yield and muscle pH

Animals were weighed prior to slaughter and then processed at the FVSU slaughter and meat processing facility. After final carcass wash, hot carcass weights were recorded. Dressed carcasses were stored at 2°C for 24 h before fabrication. After 24 h cooling, cold carcass weights were also recorded. Dressing percent of each carcass was reported as carcass yield. Muscle pH was recorded at 0-(immediately after skinning) and 24-h postmortem using a portable pH meter (Fisher Scientific, Pittsburgh, PA) with a penetrating probe (Pakton® Model OKPH1000N, Fisher Scientific). The probe was inserted directly into the *longissimus* muscle of each carcass to measure pH.

#### *Microbial counts*

Sterile sponges, hydrated with 10 mL of buffered peptone water (BioPro Enviro-Sponge Bags, International BioProducts, Redmond, WA) with disposable sterile paper templates (5 cm × 5 cm) were used for collection of skin and carcass swab samples. The swab sampling procedure for skin was adopted from Kannan et al. [26]. Samples were obtained from each animal at the beginning of the feeding trial and prior to slaughter by swabbing the hind leg within the 25 cm^2^ template area with five vertical wipes and five horizontal wipes. Carcass swab sampling was followed by the USDA procedure used for genetic *E. coli* testing [27] as modified by Kannan et al. [26]. The modification was the smaller sampling area and fewer wipes to suit the smaller size of goat carcasses instead of the 10 cm × 10 cm template recommended by the USDA. Swab samples were collected from each carcass after skinning and evisceration, but before washing by swabbing three different anatomical locations (flank, brisket, leg) within the 25-cm^2^ template area for a total sampling area of 75 cm^2^. The swab samples were placed in sterilized sponge bags, transported on ice, and stored under refrigeration until analysis.

After swabbing, the sponges were transferred into sterilized stomacher bags and 90-mL of 0.1% sterile buffered peptone water (Difco Laboratories, Detroit, MI) was added to each bag. The contents of the bag were pummeled in a stomacher (Seward Model 400, Tekmar Co. Cincinnati, OH) for 1 min. Serial dilutions were prepared with 0.1% sterile buffered peptone water. The 3M™ Petrifilm plate techniques were used to enumerate microbial loads on skin and carcass samples as recommended by the manufacturer [28]. Appropriate sample dilutions were inoculated on Petrifilm plates (3M™ Microbiology Products, St. Paul, MN) to determine *E. coli* and total coliform (3M™ Petrifilm™ *E. coli*/coliform Counts Plates) counts (TCC), and aerobic plate (3M™ Petrifilm™ aerobic Count Plates) counts (APC) as prescribed by the supplier. Colonies were counted after 24-h incubation in a Fisher Isotemp incubator (Fisher Scientific, Pittsburgh, PA) at 35°C for *E. coli* and total coliform counts, and after 48-h incubation for aerobic plate counts. Bacterial counts of skin and carcass samples were converted to log_10_ CFU/cm^2^ values.

#### *Statistical analysis*

The body weight (BW) data were analyzed as a Completely Randomized Design (CRD) with repeated measures using the PROC MIXED procedure of SAS (SAS institute Inc., Cary, NC), with individual animal as experimental unit. The effects of species, diet, FDT, and their interactions were considered to be fixed effects. Behavior data were analyzed as a CRD with 2 × 2 factorial treatment arrangement using the PROC MIXED procedures of SAS, with animal as a random effect and species, diet, and their interactions considered as fixed effects. Blood data were also analyzed as a CRD with 2 × 2 × 2 factorial treatment using the PROC MIXED procedures of SAS, with animal considered to be a random effect. The effects of species, diet, FDT, and their interactions were considered to be fixed, with pretrial concentrations as covariate. Carcass yield, muscle pH, and microbial data were also analyzed as a CRD with 2 × 2 × 2 factorial treatment using the PROC MIXED procedures of the SAS, with animals considered to be a random effect and species, diet, and feed deprivation considered to be fixed effects.

The following statistical models were used to analyze 1) body weight data; 2) behavior data (standing, moving, agonistic, lying and drinking); 3) blood data (plasma cortisol, glucose, creatine kinase, plasma urea nitrogen, and non-esterified fatty acids); and 4) carcass yield, muscle pH and microbial data (*E. coli, Enterobacteriaceae,* total coliform, and aerobic plate counts):

(1) $$\begin{array}{l} Y_{\mathrm{ijklm}}=\mu +\;S_{i}+\;D_{j}+\;F_{k}+\;T_{l}+\;{\mathrm{SD}}_{\mathrm{ij}}+\;{\mathrm{SF}}_{\mathrm{ik}}+\;{\mathrm{ST}}_{\mathrm{il}}\\ \;+{\mathrm{DF}}_{\mathrm{jk}}+\;{\mathrm{DT}}_{\mathrm{jl}}+\;{\mathrm{FT}}_{\mathrm{kl}}++\;{\mathrm{SDF}}_{\mathrm{ijk}}+\;{\mathrm{SDT}}_{\mathrm{ijl}}\\ \;+{\mathrm{SFT}}_{\mathrm{ikl}}+\;{\mathrm{DFT}}_{\mathrm{jkl}}+\;{\mathrm{SDFT}}_{\mathrm{ijkl}}+\;e_{\mathrm{ijklm}} \end{array}$$

(2) $$Y_{\mathrm{ijk}}=\mu +\;S_{i}+\;D_{j}+\;{\mathrm{SD}}_{\mathrm{ij}}+\;e_{\mathrm{ijk}}$$

(3) $$\begin{array}{c} Y_{\mathrm{ijklm}}=\mu +\;S_{i}+\;D_{j}+\;{\mathrm{SD}}_{\mathrm{ij}}+\;{\mathrm{DF}}_{\mathrm{jk}}+\;{\mathrm{SF}}_{\mathrm{ik}}\\ \;+{\mathrm{SDF}}_{\mathrm{ijk}}+\;B_{l}+\;e_{\mathrm{ijklm}} \end{array}$$

(4) $$\begin{array}{c} Y_{\mathrm{ijkl}}=\mu +\;S_{i}+\;D_{j}+\;F_{k}+\;{\mathrm{SD}}_{\mathrm{ij}}+\;{\mathrm{DF}}_{\mathrm{jk}}+\;{\mathrm{SF}}_{\mathrm{ik}}\\ \;+{\mathrm{SDF}}_{\mathrm{ijk}}+\;e_{\mathrm{ijkl}} \end{array}$$

Where Y_ijkl_ or Y_ijklm_ = dependent variables, μ = overall means, S_i_ = species, D_j_ = diet, F_k_ = feed deprivation, T_l_ = body weights (prior to diet, feed deprivation, and slaughter) as repeated measures, B_l_ = pretrial concentrations of plasma cortisol, glucose, creatine kinase, plasma urea nitrogen, or non-esterified fatty acids, e_ijkl_ or e_ijklm_ = residuals.

From each analysis, least squares means were generated and when significant by ANOVA, separated using the PDIFF option of SAS for main or interaction effects. Pearson correlation analysis (SAS Institute Inc.) was performed to study the relationships among selected dependent variables [29]. Significance was determined at *P* < 0.05, but difference of 0.05 ≤ *P* < 0.1 was considered as trends.

## Results

### Body weight and animal behavior

**Figure 1:**
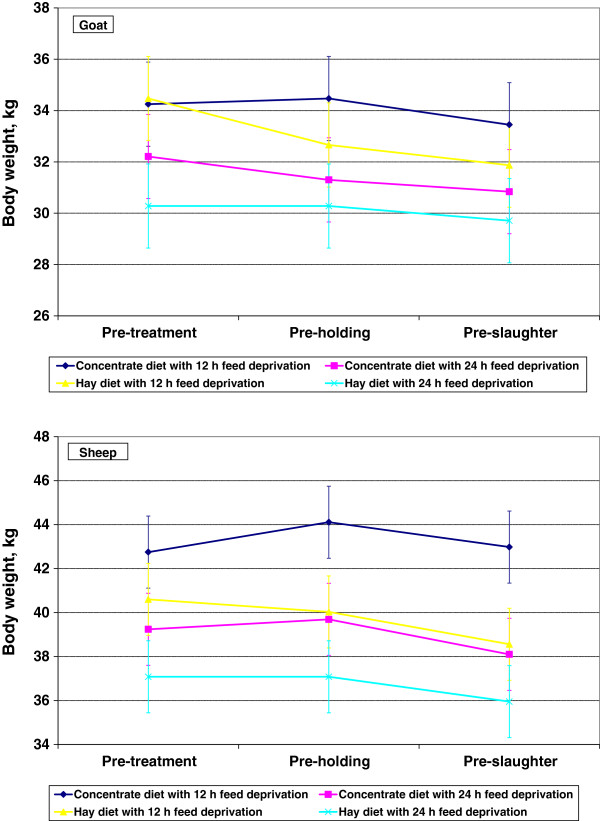
Body weights of sheep and goats measured prior to feeding (pre-treatment), holding (pre-holding), and slaughter (pre-slaughter).

The mean BW of sheep (39.6 ± 0.47 kg) was significantly higher than that of goats (32.1 ± 0.47 kg) in this experiment (Figure 1). Diet and FDT significantly influenced (*P* < 0.05) BW of experimental animals. The BW were 36.9 ± 0.47 and 34.9 ± 0.47 kg in CD and HD groups, respectively. Mean BW of animals in the 12- and 24-h FDT groups were 36.8 ± 0.47 and 35.0 ± 0.47 kg, respectively. Species × diet and diet × FDT interactions also had significant effects on BW of animals during the experimental period (Figure 1).

**Figure 2:**
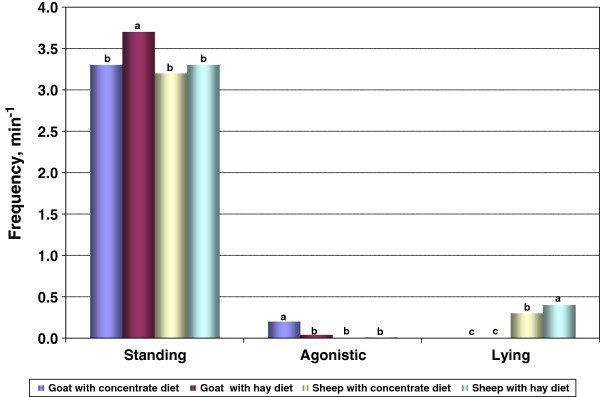
**Effect of species and diet on standing (SE = 0.076), agonistic (SE = 0.026), and lying (SE = 0.056) behaviors in the holding pens during a 90-min period prior to slaughter.** For any behavior, bars bearing different letters are different (*P* < 0.05).

Frequencies of standing, moving, and agonistic behaviors were higher (*P* < 0.05) in goats than sheep (Table 2). Sheep spent more time lying down than goats (*P* < 0.01). Animals from the CD group had significantly higher frequencies of moving and agonistic behaviors than those from the HD group. The frequencies of standing and lying behaviors were higher in animals from the HD group than those from the CD group. Animals rarely drank water during the preslaugher holding period and thus the frequency of drinking behavior was not affected (*P* > 0.05) by either species or diet. Species × diet interaction effect was significant for frequencies of standing, agonistic, and lying behaviors (Figure 2). Hay-fed goats (3.7 ± 0.08/min) had a higher (*P* < 0.05) frequency of the standing behavior compared to other treatment groups (species × diet); and, concentrate-fed goats (0.2 ± 0.03/min) also had a higher (*P* < 0.05) frequency of agonistic behavior compared to other groups. However, sheep (0.6 ± 0.06/min) fed with the concentrate had a higher (*P* < 0.05) frequency of the lying behavior compared to other groups.

**Table 2:** Effects of species, diet, and feed deprivation time (FDT) on blood hormone, metabolite, behavior, and muscle pH in sheep and goats

	**Species**			**Diet**			**FDT**			
**Response**	**Goat**	**Sheep**	***P*-value**	**HD**	**CD**	***P*-value**	**12-hrs**	**24-hrs**	***P*-value**	**SE**
**n**	**16**	**16**		**16**	**16**		**16**	**16**		
Blood hormone										
Plasma cortisol, ng/mL	54.76	80.11	0.2027	62.79	72.08	0.6452	64.84	70.02	07911	13.612
Blood metabolite^1^										
Plasma glucose, mg/dL	130.39	128.54	0.8888	122.60	136.24	0.2159	132.37	126.47	0.5888	7.551
PUN, mg/dL	17.24	18.30	0.6820	17.24	18.30	0.6468	17.16	18.38	0.5944	1.603
Plasma CK activity, IU	571.74^b^	863.94^a^	0.0392	732.97	702.72	0.8265	747.15	688.54	0.6798	95.210
Plasma NEFA, mEq/L	589.78^b^	1056.05^a^	0.0054	953.56	692.27	0.1105	729.05	916.79	0.2108	105.01
Behavioral observations^2^										
Standing	3.49^a^	3.24^b^	0.0008	3.46^a^	3.26^b^	0.0084				
Moving	0.37^a^	0.28^b^	0.0440	0.19^b^	0.46^a^	0.0001				
Agonistic	0.13^a^	0.01^b^	0.0001	0.03^b^	0.11^a^	0.0024				
Lying	0.00^b^	0.48^a^	0.0001	0.32^a^	0.16^b^	0.0043				
Drink	0.00	0.003	0.3176	0.002	0.00	0.3176				
Dressing percent,%	43.02	42.84	0.7380	42.59	43.28	0.2049	42.79	43.07	0.5959	0.3752
Muscle pH^3^										
Initial	6.96	6.96	0.6000	6.97	6.91	0.2740	6.92	6.97	0.3688	0.039
Ultimate	6.02^a^	5.84^b^	0.0002	5.98^a^	5.87^b^	0.0141	5.92	5.93	0.8678	0.029

HD = hay diet; CD = concentrate diet.

^1^PUN = plasma urea nitrogen; CK = creatine kinase; NEFA = non-esterified fatty acids.

^2^Observed or 90 min before staring the slaughtering process.

^3^Inital = pH of *longissimus* muscle at immediately after skinning; Ultimate = pH of *longissimus* muscle at 24 h postmortem.

^a,b^Within a row, least squares means that do not have a common superscript letter differ (*P* < 0.05).

### Blood hormone and metabolite concentrations

Plasma cortisol, glucose, and plasma urea nitrogen (PUN) concentrations were not influenced (*P* > 0.05) by any of the factors studied (Table 2). The interaction effects were also not significant for plasma cortisol or any of the metabolic concentrations (glucose, creatine kinase, plasma urea nitrogen, and non-esterified fatty acids). However, plasma creatine kinase (CK) activities and non-esterified fatty acids (NEFA) levels were different (*P* < 0.05) between species (Table 2). The HD animals tended to have higher (*P* = 0.11) plasma NEFA levels than CD animals (Table 2).

### Carcass yield and muscle pH

**Figure 3:**
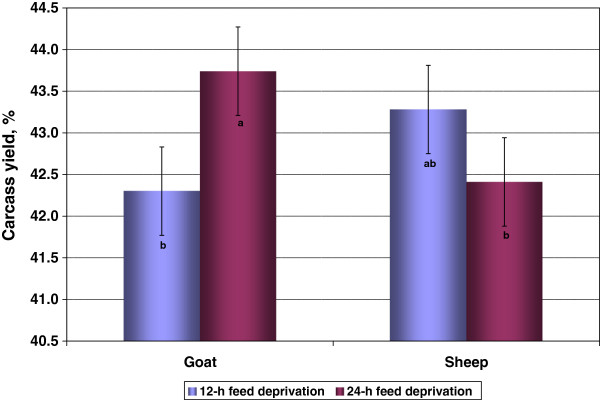
**Effect of species and feed deprivation time on carcass yields (SE = 0.531).** Bars bearing different letters are different (*P* < 0.05).

Carcass yield ranged from 40 to 45% in the present experiment, but was not influenced by species, diet, or FDT (Table 2). However, goats (43.7 ± 0.53%) deprived of feed for 24-h had a higher mean carcass yield than those deprived for 12-h (42.3 ± 0.53%), while an opposite trend was noticed in sheep (species × FDT, *P* < 0.05, Figure 3).

**Figure 4:**
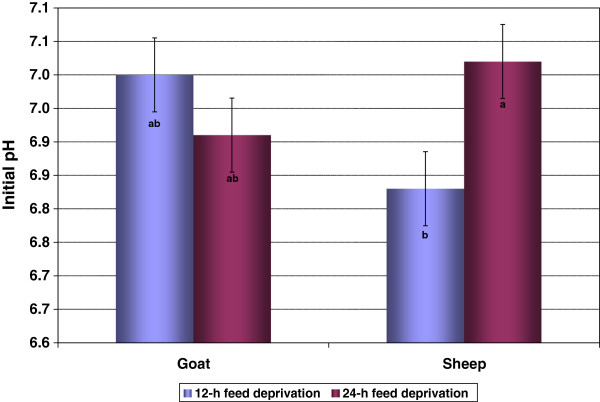
**Effect of species and feed deprivation time on the initial pH of *longissimus *muscle (SE = 0.055).** Bars bearing different letters are different (*P* < 0.05).

The initial pH of LM was not affected (*P* > 0.05) by species, diet, or FDT (Table 2). However, species x FDT interaction effect was significant (Figure 4). Sheep subjected to 12-h feed deprivation (7.02 ± 0.055) had lower (*P* < 0.05) pH values than those subjected to 24-h feed deprivation (6.83 ± 0.055), while the initial pH of goat carcasses were not affected by FDT. The ultimate pH was higher in goats compared to sheep (*P* < 0.05) and higher in HD compared to CD animals (*P* < 0.05, Table 2). However, the interaction effects were not significant (*P* > 0.05) for the ultimate muscle pH values in the current study.

### Skin bacterial counts

**Figure 5:**
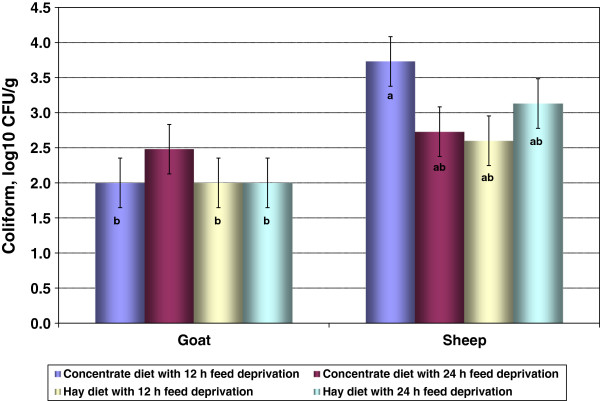
**Effect of species, diet, and feed deprivation time on pre-holding total coliform counts of skin swab samples (SE = 0.353).** Bar bearing different letters are different (*P* < 0.05).

Pre-holding *E. coli* counts of skin samples were not influenced (*P* < 0.05) by species, diet, or diet × species interaction effects (Table 3). However, TCC of skin were significantly different between the two species. Goats had lower (*P* < 0.05) coliform counts in the skin than sheep (Table 3). Diet treatments or diet x species interaction factors had no significant effects on TCC (Table 3). The TCC was influenced (*P* < 0.05) by the species × diet × FDT interaction (Figure 5), with concentrate-fed sheep having the highest TCC (*P* < 0.05, 3.73 ± 0.353 log_10_ CFU/cm^2^) after 12 h feed deprivation compared to all other groups. Goats also had lower (*P* < 0.05) skin APC compared to sheep (Table 3). No significant effects of diet and diet x species interaction were detected in aerobic plate counts of skin.

**Table 3:** **Effects of species, diet, and feed deprivation time (FDT) on the microbial counts (log**_**10 **_**CFU/cm**^**2**^**) on skin and carcass of sheep and goats**

	**Species**			**Diet**			**FDT**			
**Response**	**Goat**	**Sheep**	***P*-value**	**HD**	**CD**	***P*-value**	**12-hrs**	**24-hrs**	***P*-value**	**SE**
**n**	**16**	**16**		**16**	**16**		**16**	**16**		
Skin, pre-holding^1^										
*E. coli* count	2.00	2.21	0.2129	2.06	2.16	0.5527				
Total coliform count	2.12^b^	3.04^a^	0.0011	2.43	2.73	0.2413				
Aerobic plate count	8.21^b^	8.53^a^	0.0305	8.42	8.31	0.4511				
Skin, pre-slaughtering^2^										
*E. coli* count	2.23^b^	2.93^a^	0.0118	2.56	2.60	0.8650	2.38	2.78	0.1238	0.180
Total coliform count	2.26^b^	3.01^a^	0.0135	2.60	2.67	0.8074	2.42	2.85	0.1352	0.197
Aerobic plate count	8.31	8.30	0.8017	8.33	8.28	0.3198	8.34	8.28	0.2163	0.035
Carcass, pre-washing^3^										
*E. coli* count	2.28	2.51	0.2212	2.34	2.44	0.6018	2.28	2.41	0.8959	0.134
Total coliform count	2.29	2.56	0.1999	2.35	2.50	0.4587	2.43	2.43	1.0000	0.141
Aerobic plate count	7.93	8.28	0.0718	8.16	8.04	0.5076	8.35^a^	7.85^b^	0.0128	0.131

HD = hay diet; CD = concentrate diet.

^1^Skin swabs from hind leg before depriving feed to experimental animals.

^2^Skin swabs from hind leg right before starting slaughtering.

^3^Carcass swabs from flank, brisket and leg regions right before washing after skinning and gut removed.

^a,b^Within a row, least squares means that do not have a common superscript letter differ (*P* < 0.05).

Preslaughter skin *E. coli* counts and TCC were different (*P* < 0.05) between species (Table 3), and goats had lower (*P* < 0.05) counts of *E. coli* and TCC in skin swab samples compared with sheep. Diet, FDT, or interaction effects were not significant (*P* > 0.05) for skin *E. coli* and total coliform counts (Table 3). However, skin swab samples of the 24-h feed deprivation group tended (*P* = 0.12) to have higher *E. coli* counts than the 12 h group. Total coliform counts tended (*P* = 0.14) to be higher in the 24-h group than 12-h feed deprivation group (Table 3). Aerobic plate counts of skin swab samples were not influenced (*P* > 0.05) by any of the main effects or interactions.

### Carcass bacterial counts

Diet, species, FDT, and their interactions had no significant effects on *E. coli* and coliform counts (Table 3). Aerobic plate counts of carcass swab samples were also not influenced (*P* > 0.05) by any of the treatment factors or their interactions, except the feed deprivation (FD) time (Table 3). Carcasses from the 12 h feed deprivation group had higher (*P* < 0.05) APC than those from 24 h group. Carcass swab samples from sheep tended (*P* = 0.07) to have higher APC than those from goats (Table 3).

## Discussion

In sheep, the body weights appeared to increase due to concentrate feeding, but did not change or decrease due to hay feeding. In goats, there was no clear pattern in body weight changes due to diet. However, body weights decreased due to feed deprivation in both sheep and goats. Live weight losses during the preslaughter period are of major concern in small ruminants. Live weight shrinkage can be about 10% in goats after 18 h feed deprivation, and about 7% in sheep after 24 h feed deprivation [12,13]. These live weight losses can be attributed to reductions in gut weights, since the gastrointestinal tract contributes to a major proportion of live weight in small ruminants [30].

Plasma cortisol and certain metabolite concentrations are good indicators of the physiological status of animals as influenced by preslaughter dietary treatment and FDT. The cortisol concentrations were not influenced by species, diet, or FDT in the present experiment. Feed deprivation combined with a 2.5-h transportation has been reported to elevate cortisol concentrations in goats [12]. Transporting animals from the experimental facility to the slaughter plant was completed within 10 min in the present study. It appears that feed deprivation alone for 12 or 24 h is not stressful enough to elevate circulating cortisol concentrations in sheep and goats. A similar effect was observed in a previous study when Spanish does were feed deprived for 7, 14, or 21 h [24]. However, feed deprivation has been reported to elevate cortisol concentrations in sheep [19]. Creatine kinase activities were higher in sheep compared to goats, although behavioral observations showed that goats were more active than sheep during preslaughter holding. Creatine kinase activity in blood increases due to muscle damage or increased muscular activity in animals [31]. Plasma NEFA concentrations were also higher in sheep than goats. The higher CK and NEFA levels may be attributed simply to a species difference. It is not clear if size of animals could have contributed to this effect, since the sheep used in this study were heavier than goats. Hay-fed animals tended to have higher NEFA concentrations than concentrate-fed animals. Furthermore, animals subjected to 24 h of feed deprivation tended to have higher NEFA concentrations than those subjected to 12 h of feed deprivation. Knowles et al. [22] reported that the plasma NEFA concentrations increased in sheep after 24 h feed deprivation. Kouakou et al. [23] found that feed restriction elevated plasma NEFA concentration in goats because feed deprivation increases lipolysis in animals, which in turn increases free fatty acid levels in the blood [32].

In the present study, carcasses from CD group had lower ultimate LM pH values than HD animals (Table 2). Variation in glycogen content of muscles may be responsible for the differences in the ultimate pH. Glycogen content of muscles at the time of slaughter is the important factor that affects muscle ultimate pH and meat quality [33]. Forage-finished animals have been reported to produce lower quality meat than grain-finished animals [34,35]. Immonem et al. [36] reported variations in the ultimate pH of muscle due to energy levels in the diet of cattle, and they found that cattle fed a high energy diet had a lower (*P* < 0.05) ultimate muscle pH (5.69 ± 0.03) value compared to cattle fed a low energy diet (5.93 ± 0.03). In contrast, Diaz et al. [37] did not find any significant differences in meat quality and muscle pH (measured immediately after slaughter, after 45 min and after 24 h) from pasture-fed and concentrate-fed lambs.

The skin of a live animal becomes contaminated with microorganisms derived from a wide range of sources such as feces, soil, water and vegetation [2]. The APC, TCC, and *E. coli* counts of skin swab samples collected at two different times in the present study was not influenced by diet. Skin swab samples collected from sheep showed higher bacterial counts than goats. Sheep fleece may be responsible for picking up fecal material from the pen floor and retaining the contamination for longer time. Behavioral observations of the animals during holding period revealed that sheep tended to spend more time lying down in the pens than goats. It is possible that goats were not able to withstand the cold temperature of concrete floors, which would have prevented them from lying down. This experiment was conducted in the month of December. Skin swab samples collected from the 24-h feed deprivation group showed higher *E. coli* counts than the 12-h group. There is always more chance for skin contamination with fecal material if the animals spend more time in the holding pens.

Preslaughter diet and feed deprivation time had no effect on TCC and *E. coli* counts of carcass swab samples. Major sources of carcass contamination are unclean animal skin and viscera of animals entering the slaughter facility [1]. Carcass TCC and *E. coli* counts were not correlated with skin counts. Elder et al. [38] found no correlation between the prevalence of *E. coli* O157 contamination on cattle hides and that resulting on carcasses. In their study, the prevalence of *E. coli* O157 on the carcasses was higher than that on hides.

## Conclusions

Preslaughter diet and FDT did not influence the physiological status of sheep and goats according to plasma cortisol, glucose, CK, PUN, and NEFA. Feed deprivation may significantly decrease body weights in sheep and goats. Sheep had higher skin contamination than goats, probably due to differences in their behavior during preslaughter holding. Sheep spent more time lying down than goats in holding pens. Diet and FDT did not influence skin contamination in sheep and goats. There was no relationship between skin contamination and carcass contamination in the present study. Preslaughter diet may have an effect on the energy reserves in muscles, as the LM ultimate pH was lower in the concentrate-fed group. The results indicate that diet can be manipulated without significant effects on physiological responses in sheep and goats.

## Competing interests

The authors declare that they have no competing interests.

## Authors’ contributions

All authors made significant contributions to design and perform the research. Especially GK and VRG conducted all data analyses and drafted the initial manuscript. All authors read and agreed the final manuscript.
